# Current Approach in Surface Plasmons for Thin Film and Wire Array Solar Cell Applications

**DOI:** 10.3390/ma8074565

**Published:** 2015-07-22

**Authors:** Keya Zhou, Zhongyi Guo, Shutian Liu, Jung-Ho Lee

**Affiliations:** 1 Department of Physics, Harbin Institute of Technology, Harbin 150001, China; E-Mails: zhoukeya@hit.edu.cn (K.Z.); stliu@hit.edu.cn (S.L.); 2 School of Computer and Information, Hefei University of Technology, Hefei 230009, China; E-Mail: zyguo@hit.edu.cn; 3 Department of Materials and Chemical Engineering, Hanyang University, Ansan, Kyounggi 426-791, Korea

**Keywords:** surface plasmons, silicon, silicon nanowire, solar cell, thin film solar cells, organic solar cells, optical losses, light scattering, optical trapping, nanoparticles, photovoltaics, hot electron, guide mode

## Abstract

Surface plasmons, which exist along the interface of a metal and a dielectric, have been proposed as an efficient alternative method for light trapping in solar cells during the past ten years. With unique properties such as superior light scattering, optical trapping, guide mode coupling, near field concentration, and hot-electron generation, metallic nanoparticles or nanostructures can be tailored to a certain geometric design to enhance solar cell conversion efficiency and to reduce the material costs. In this article, we review current approaches on different kinds of solar cells, such as crystalline silicon (c-Si) and amorphous silicon (a-Si) thin film solar cells, organic solar cells, nanowire array solar cells, and single nanowire solar cells.

## 1. Introduction

Surface plasmons (SPs) are collective oscillations of free electrons localized at the interfaces of a metal and a dielectric. The origin of SPs can be traced back to the early 20th century. In 1902, Wood [1] reported an anomalous decrease in the intensity of light reflected by a metallic grating, which could be attributed to the excitation of SPs mediated by the periodic structure of the grating. The current electromagnetic theory of SPs is based on two pioneering works. The first one is the theory of light scattering and absorption by spherical particles developed by Mie [2] in 1908. The second one is the dispersion relationships of SPs in metallic films as derived by Ritchie [3] in 1957. Essentially, SPs are light waves that are trapped on the surface of metals due to their interaction with the free electrons of the metals. In this interaction, the free electrons respond collectively by oscillating in resonance with the light wave. Such a resonant interaction constitutes SPs and gives rise to their unique properties [4,5].

Since 1990, the field of surface plasmons has become application-driven, and started to be researched in optical emitters, plasmon focusing, nanoscale optical antennas, plasmonic integrated circuits, nanoscale switches, plasmonic lasers, light-emitting diodes, imaging below the diffraction limit, materials with negative refractive index and so on [6,7,8,9,10,11,12]. Beginning in 2005, researchers turned their attention to the applications in photovoltaics [13].

One of the great challenges facing society today is the need for low-cost, environmentally friendly energy sources that can meet the growing demands of an expanding population. Solar energy has the potential to meet such requirements. However, compared to other renewable and non-renewable energy resources, the cost of photovoltaic modules is high, which constrains their applications. To date, most commercial photovoltaic cells have been based on silicon wafers. As crystalline Si (c-Si) is a weak absorber, especially at infrared wavelengths close to its bandgap, it usually requires the thickness of more than 300 μm in semiconductor material to fully absorb incident sunlight. Although thin-film photovoltaic cells of a few micrometers in thickness with organic or inorganic semiconductors have been developed as an alternative, the conversion efficiency of such thin film solar cells is still unable to outperform that of commercial crystalline silicon solar cells (~20%). Theoretically, third generation photovoltaic cells have a higher efficiency with lower cost, but sufficient photon harvesting is required in solar cells, in which the SPs can act for achieving this purpose. The beneficial effects associated with surface plasmon resonance in photovoltaics include superior light scattering by metallic nanoparticles, enhancement of the local field in semiconductors, optical coupling of the incident light to waveguide modes, as well as hot-electron generation in metallic nanoparticles [13,14,15,16,17,18,19]. Since SPs are highly sensitive to particle shapes, sizes, material properties, and surrounding environments, the adoption of SPs in solar cells can be realized in various ways. In this article, the current status of development of SPs into a variety of solar cells is summarized.

## 2. Surface Plasmonics in Thin Film Solar Cells

Both inorganic [20,21,22,23,24,25,26,27,28,29] and organic [30,31,32,33,34,35,36,37] thin film solar cells can utilize SPs to enhance their conversion efficiency. As for c-Si thin film solar cells with a thickness of 2–3 μm, incident light cannot be fully absorbed especially in wavelengths close to silicon bandgap. As for a-Si:H thin film solar cells, although its absorption coefficient is much higher than that of c-Si, a carrier diffusion length in a-Si:H is only on the order of 100 nm, which is much shorter than that in c-Si. The thickness of the active layer must be less than 1 μm in order to facilitate carrier transport before the free carriers recombine. As a result, light trapping is also essential in a-Si:H thin film solar cells to the increase the light absorption. In both cases, different surface textured transparent conductive oxides (TCOs) have been used to increase the light trapping in active layers. Alternatively, SPs provide another light trapping route without using such textured surfaces, which then attracted a lot attention [18]. As for organic solar cells, the charge collection efficiency decreases as the thickness of the active layer increases, the active layer is often as thin as 100–200 nm. Due to their micron-scale thickness, traditional methods of increasing light absorption based upon surface textures may be unreasonable. Alternatively, SPs could be adopted into such organic solar cells for enhancing light absorption. In general, SPs can take the form of nanoparticles or nanostructures, which will be reviewed separately in the following subsections.

### 2.1. Plasmonic Nanoparticle-Based Thin Film Solar Cells

**Figure 1:**
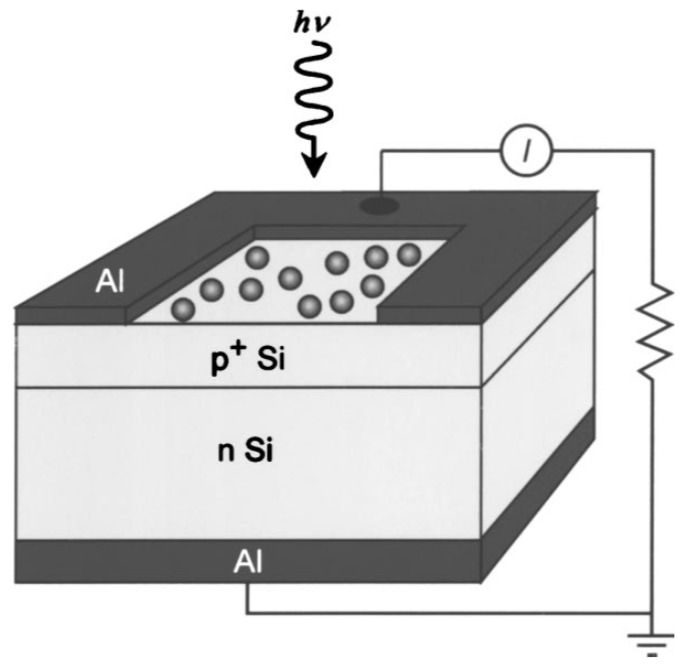
Schematic diagram of Si p–n junction diode device structure with metallic nanoparticles. Reprinted with permission from [13]. Copyright © 2005 American Institute of Physics.

Early in 2005, Schaadt *et al.*, reported an engineered enhancement of absorption and photocurrent in a semiconductor via excitation of surface plasmon resonances in Au nanoparticles (NPs) deposited on a semiconductor surface [13]. The basic device structure is shown schematically in Figure 1. Au NPs with diameters of 50, 80, and 100 nm are compared, and the surface coverages range from 0.6% to 1.3%. It was concluded that for a p–n junction diode, increased optical absorption due to the presence of metal nanoparticles was manifested as an increase in photocurrent response at wavelengths corresponding to those of the nanoparticle surface plasmon resonances. In 2007, Pillai *et al.*, investigated the effect of Ag NPs for enhancing the absorbance of thin film c-Si solar cells [20]. In their study, NPs had much smaller diameters (less than 30 nm). Their pioneering findings showed that for front surface application, smaller metal NPs provided the maximum overall enhancement in visible light as well as the near- infrared for solar cell applications, but that larger metal NPs would be more beneficial for light emission from both thin and thick Si light emitting diodes (LEDs).

In 2008, Catchpole *et al.*, theoretically studied the light scattering from a single Ag or Au particle, varying the shape, size, particle material, and dielectric environment [21]. They showed that cylindrical and hemispherical NPs could lead to much higher path length enhancements than spherical NPs. Ag NPs caused much higher path length enhancements than Au NPs. The distance of NPs from the substrate is an important factor in absorption enhancement, which is related to the excitation of gap modes inside the solar cell device [22,23,24]. In 2009, Akimov *et al.*, used predictive 3D modeling to investigate the effect of higher-order modes on that enhancement [25]. They performed both size and coverage optimization, and proposed two optimal configurations of Ag NPs with diameters of 30 nm and 80 nm. The optimal coverage was suggested to be around 33% and 11%, respectively. In 2011, Spinelli *et al.*, systematically studied the coupling of light into a crystalline silicon substrate by scattering light from Ag NP array geometries [26]. After simulation and optimization, spheroidal Ag NPs 200 nm wide and 125 nm high in a square array with 450 nm pitch on top of a 50-nm-thick Si_3_N_4_ layer provided the best impedance matching for a spectral distribution corresponding to the Air Mass 1.5 solar spectrum.

**Figure 2:**
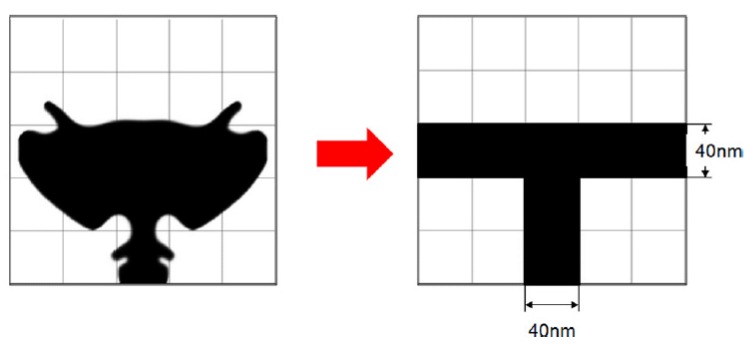
Simplified nanostructure with T-profile derived from the nanoparticle shape. Reprinted with permission from [27]. Copyright © 2014 AIP Publishing LLC.

In 2014, Byun *et al.*, suggested a parabolic antenna-type Ag NP shape, and confirmed that its simplified shape (a T-profile) enhanced the field intensity of the absorbing layer in a visible wavelength range, especially over 650 nm [27]. The shapes of the derived optimal and simplified NPs are schematically shown in Figure 2. The above mentioned theoretical work mostly provides valuable suggestions on optimizing metallic NPs in both a-Si and c-Si thin film solar cells. The optimized geometries of metallic NPs should consider particle sizes, shapes, relative positions, coverage, material types, local dielectric environment, and so on. Nonetheless, those findings may lack in experimental verifications. In the year 2013, an experimental work by Park *et al.*, achieved the increased photocurrent of ~45% in polycrystalline silicon thin film solar cells by using optimized plasmonic Ag NPs. Their absolute efficiency of 5.32% (without a back reflector) and 5.95% (with the back reflector) are the efficiency for metallized plasmonic solar cells highest reported to date [28,29].

Metallic NPs can also be implemented in organic thin film solar cells, in which NPs are practically placed either into the active (or buffer) layer or at various interfaces [30]. Regardless of their electrical properties, organic thin film solar cells are much beneficial for intriguing optical properties of metallic NPs. For example, small NPs with diameters in a range of 5–20 nm behave as sub-wavelength antennas, coupling near-field into the active layer; NPs with relatively larger diameters (>50 nm) could behave as effective sub-wavelength scattering elements, coupling and trapping incident freely propagating light into the active layer. Furthermore, if NPs are placed in a periodically arranged array, incident light could be efficiently coupled into waveguide modes of the active layer under the condition of momentum matching [4]. In general, metallic NPs can take the shapes of nanoclusters [31], nanoislands [32], nanodisks [33], nanospheres [34,35], nanoplates [36], nanowires [37], and so on. Tuning the plasmon resonance is critically required because the metal types, NP sizes, shapes, and their positions are variable with the thickness of active layer. The electrical properties of the devices need to be also considered. Yoon *et al.*, have recorded an increased photocurrent by placing a self-assembled monolayer of Ag nanospheres between the poly (3,4-ethylenedioxythiophene) poly (styrenesulfonate) (PEDOT:PSS) and the photoactive layer of P3HT:PCBM cells [34]. However, significant losses of open-circuit voltage (Voc) and fill factor (FF) were observed, implying the possibility of Ag NPs acting as an energy barrier for the charge extraction and injection. Actually, revealing their exact role on solar cell performance remains challenge because the electrical properties of NP-based organic solar cells are much complicated to understand.

### 2.2. Plasmonic Nanostructure-Based Thin Film Solar Cells

**Figure 3:**
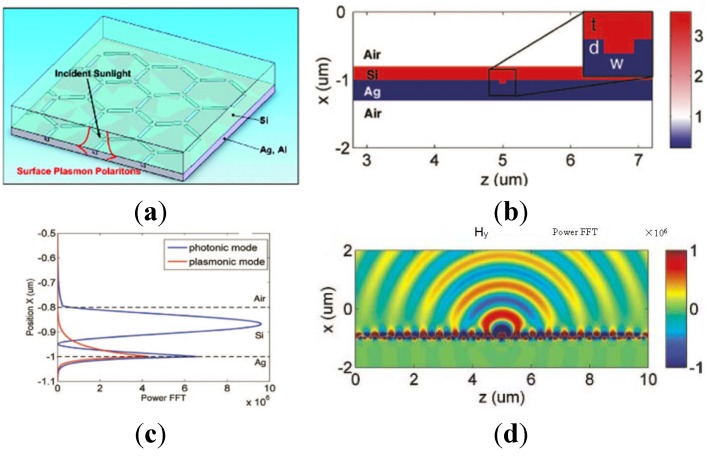
(**a**) A 3D conceptual schematic of a solar cell with subwavelength grooves tiled on a back Ag contact. (**b**) The schematic outline of the device layer in the simulation, with the inset of panel b defining the width and depth of a groove. (**c**) The modal profiles of the two identified modes, plotting power as a function of position in the waveguide. (**d**) The scattered transverse magnetic field Hy of a 100-nm-wide by 50-nm-deep groove filled with Si. Reprinted with permission from [38]. Copyright © 2008 American Chemical Society.

**Figure 4:**
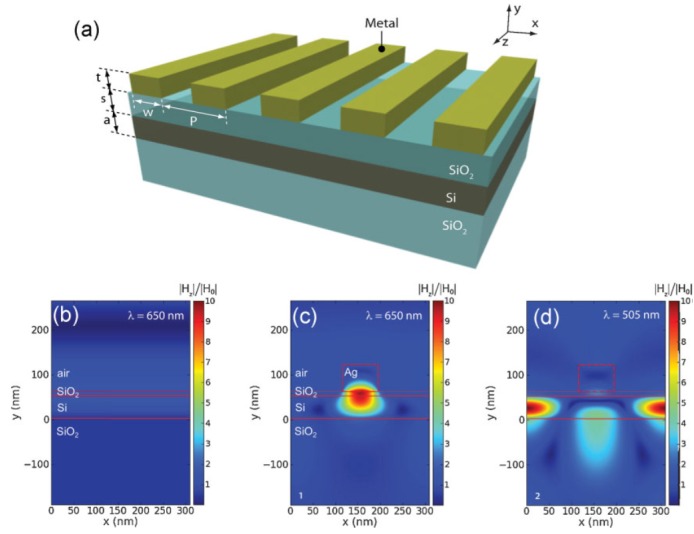
(**a**) Schematic showing the proposed plasmon-enhanced cell structure. Normalized and time-averaged field intensity plots for normal incidence. (**b**) TM illumination of a bare Si/SiO_2_ structure, and (**c**,**d**) The same structure with a periodic array of metal strips spaced at p = 312 nm, a spacer layer thickness of s = 10 nm, and an absorbing Si film thickness, a = 50 nm. The incoming wavelengths (energies), λ = 650 nm (1.91 eV) for panels b and c, and λ = 505 nm (2.46 eV) for panel d, which were chosen to demonstrate the effects of strong near-field light concentration or excitation of waveguide modes by the strips. Reprinted with permission from [39]. Copyright © 2009 WILEY-VCH Verlag GmbH & Co. KGaA, Weinheim.

Besides metallic NPs, two-dimensional metallic nanostructures have also been used [38,39,40,41,42]. In 2008, Ferry *et al.*, reported their findings that subwavelength scatterers can couple sunlight into guided modes in thin film Si and GaAs solar cells using a back interface coated with a corrugated metal film [38]. The schematics are shown in Figure 3. The sub-wavelength grooves on the underlying plasmonic back-contact are purposely designed with different tilted angles, which renders them remarkably insensitive to incident angles. These geometrical configurations for solar cells can be spectrally tuned through modifications of the scatterer shape, a semiconductor film thickness, and material choice. Up to 2.5-fold enhancement of light absorption has been observed at the spectrum range near the Si bandgap. Pala *et al.*, reported in 2009 that they could simultaneously take advantage of both high near-field concentration close to their SPs resonance frequency and effective coupling to waveguide modes of the semiconductors through an optimization of the Ag strip geometries [39]. A 43% enhancement in the short circuit current as compared to a cell without metallic structures was obtained. Figure 4a demonstrates the schematic of the proposed plasmon-enhanced cell structure, and Figure 4b–d displays the time-averaged field intensity plots for normal incident transverse magnetic (TM) polarized illumination with typical geometries. Such Ag strip arrays could be easily formed and the optimized structure could be obtained through calculations based on different thickness of active semiconductor layers. In 2010, Munday *et al.*, combined plasmonic gratings with traditional antireflection coatings together, and found that the optimized integrated structure can result in a 1.8-fold total integrated current improvement under AM 1.5G solar illumination [40].

**Figure 5:**
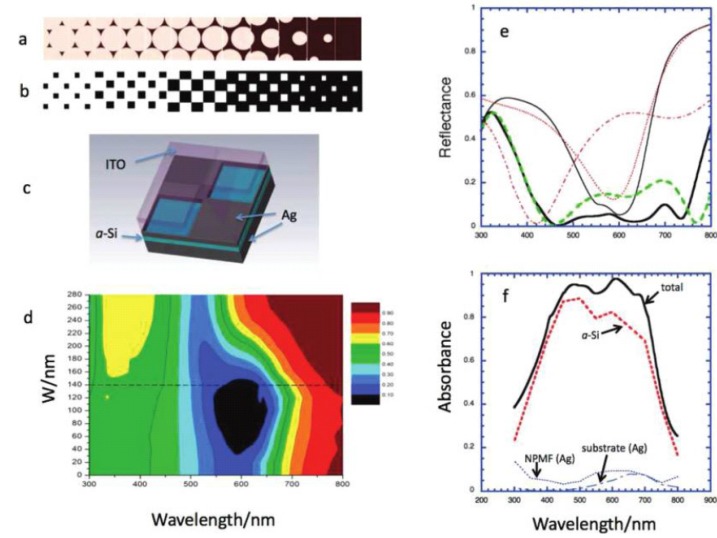
(**a**) Structures evolving from honeycomb arrays of quasi-triangular islands, to hexagonal arrays of circular holes, to a solid film. (**b**) Checkerboard series. (**c**) The in-plane unit cell of our optimized checkerboard super-absorber structure. (**d**) The color-encoded map of R *vs* W and λ. (**e**) and (**f**) are the reflectance and total absorbance spectrum. Reprinted with permission from [42]. Copyright © 2011 American Chemical Society.

Plasmonic back contacts with non-ordered Ag nanostructures for light trapping in thin-film silicon solar cells have also been studied [41]. The prepared Ag back contacts exhibited Ag nanostructures with base radius distribution maxima between 30 and 500 nm. Based on their experiments and simulation, the authors claimed that diffuse reflectance in their fabricated solar cells was caused by plasmon-induced light scattering at individual nanostructures, rather than collective effects such as diffraction as reported for plasmonic gratings. In 2011, Wang *et al.*, proposed a metamaterial plasmonic absorber structure that could be used in amorphous silicon solar cells (Figure 5) [42]. They showed that a structure consisting of a multilayer stack deposited on a metallic substrate could be made to super-absorb electromagnetic radiation in the entire visible range. Specifically, one of the layers in the stack was a nanoscopically perforated metallic film of a patterned checkerboard structure, which could function as a window electrode in the photovoltaic device. The nanoscopically perforated metallic film and the ultrathin absorber could form a metamaterial effective medium showing negative refraction in the frequency range of interest. The efficiency of the proposed specific structure should exceed 12% in practice using only 15 nm a-Si layer as absorbers.

Although SPs using metallic NPs or other nanostructures could theoretically improve the light absorption in both inorganic and organic thin solar cells compared to cases without a light trapping design, the optical losses inside metals are unavoidable. Early in 2004, Springer *et al.*, studied the absorption losses at a nanoscale rough silver back reflector of a solar cell by photothermal deflection spectroscopy [43]. The observed increase in absorption compared to the smooth silver could be explained by surface plasmon absorption. This phenomenon was further explained by Paetzold *et al.*, in 2010 through simulations with a three-dimensional numerical solver of Maxwell’s equations [44]. In 2011, Feltrin *et al.*, studied light scattering and parasitic absorption in thin film silicon solar cells containing metal nanoparticles [45]. The loss of quantum efficiency for tandem devices with metal NPs originated mainly from parasitic absorption and not from charge collection losses. Thus, an optimized plasmonic thin film solar cell must take this parasitic loss into consideration.

## 3. Surface Plasmonics in Wire-Array Solar Cells

Semiconductor wire array-based solar cells, both with microwires and nanowires, have been proven to be promising candidates for next-generation photovoltaic devices [46,47,48,49]. Wire array-based solar cells have been fabricated using c-Si, a-Si, GaAs, III-nitride, and InP via a variety of growth techniques. Compared to planar wafer-based solar cells or thin film solar cells, wire array-based solar cells have many advantageous optical and electrical properties, including reduced reflection, extreme light trapping, improved band gap tuning, facile strain relaxation, and increased defect tolerance [47]. Furthermore, they reduce the requirement for semiconductor material quantity and quality and thus reduce solar cell cost. For example, a power conversion efficiency of 4.4% has been achieved by fabricating multicrystalline Si NWs on glass in 2009, which has shown great potential for low cost solar cells [50]. The efficiency has reached 10% for polycrystalline Si NWs with a thickness of only 8 μm formed on glass by proper engineering the wires and designing the solar cell structures [51]. However, the potential of Si wire-arrays is far beyond this limit, which needs to be further exploited. In this section, we do not discuss their synthesis or fabrication, but focus on their optical properties, especially when SPs are involved.

In 2007, Hu *et al.*, predicted the optical absorption in a Si nanowire array and showed the effects of wire diameter, length, and filling ratio [52]. They concluded theoretically that nanowire structures have the advantage of low reflection over a wide spectrum range, which can be achieved without specially designed antireflection coatings. Si microwire-array solar cells with efficiencies of up to 7.9% have been fabricated using an active volume of Si equivalent to a 4-mm-thick Si wafer by Putnam *et al.*, in 2010 [53]. Usually, a metal back reflector is used as the backside of the wires in order to prevent the escape of incident illumination. Hu *et al.*, predicted an efficiency enhancement from 12.5% to 16.09% with a perfect reflecting mirror (100% reflectivity) on the backside of an 80-nm nanowire structure. Often in experiments a layer of optical thick aluminum or silver back reflector is introduced.

**Figure 6:**
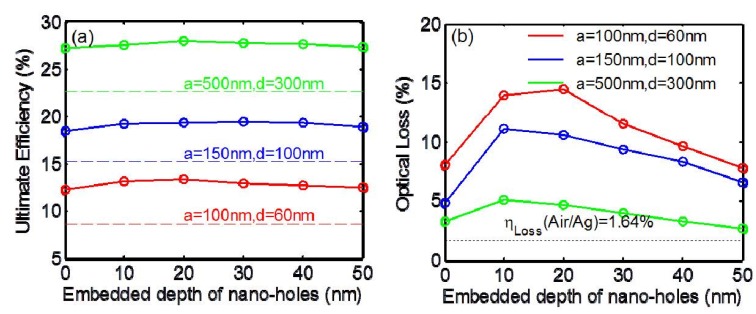
(**a**) Ultimate efficiency as a function of embedded depth into silver back reflectors (Ag BRs). (**b**) Optical losses by Ag BRs. Reprinted with permission from [55]. Copyright © 2012 Optical Society of America.

In 2010, Park *et al.*, reported a combined wire-embedded film integrated with an Al back reflector and obtained average absorption of ~91.5% for the entire spectral range of 300–1100 nm, along with a remarkable enhancement (~80) in near-infrared absorption [54]. Such an enhancement in absorption is assumed to be an SP effect due to the nanostructured Al gratings. However, similar to the case of thin film solar cells, the intrinsic absorption loss in metals should not be neglected. In 2012, we performed a systematic numerical study to characterize the tradeoffs between plasmonic enhancement and optical loss in periodically aligned, silicon nanowire (Si NW) arrays integrated with a silver back reflector (Ag BR) [55]. We assumed that the bottoms of a NW array were embedded into the Ag BR, forming a nanohole grating structure that matched the experimental results. Figure 6 demonstrates the variation in the cell ultimate efficiencies and optical losses in Ag BR for typical embedding depths ranging from 0 to 50 nm. After simulation, it was discovered that, although the plasmonic loss in the back reflector is higher than that in a thin film solar cell, it could be helpful for efficiently promoting the efficiencies after integrating the wires into an Ag BR with an optimal embedded wire depth of ~20 nm. Such a simulation result proves that a nano-hole array back contact could be more efficient than a flat back reflector in Si NW array solar cells through the excitation of localized surface plasmons and guided modes in Si wires.

**Figure 7:**
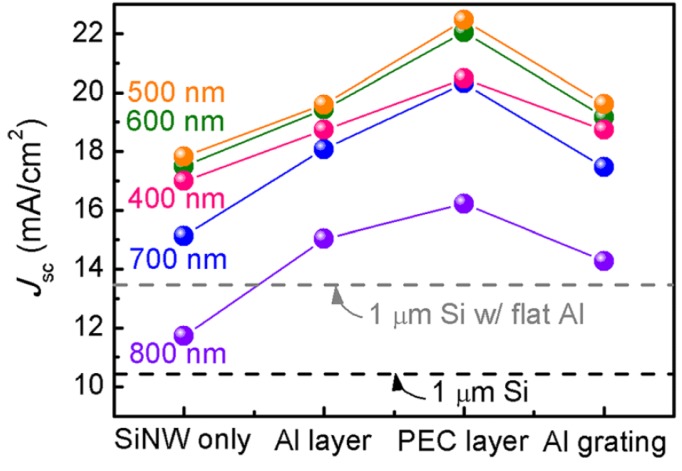
Calculated *J*_sc_ for several types of 400-nm-diameter nanowire (NW) arrays with various periods (400–800 nm). Gray and black dashed lines indicate *J*_sc_ of the 1-μm-thick Si with and without the flat Al underlayer, respectively. Reprinted with permission from [56]. Copyright © 2013 AIP Publishing LLC.

In 2013, Lee *et al.*, studied the optical characteristics of Si NW arrays with and without Al underlayers via simulation [56]. They considered four types of Si SW arrays: Si NWs without any underlayer, Si NWs with a flat Al underlayer, Si NWs with a perfect electrical conductor (PEC) underlayer, Si NWs with an Al grating underlayer. The maximum achievable short-circuit current density (*J*_sc_) was estimated, which is shown in Figure 7, for 400-nm-diameter Si NW arrays with various periods (400–800 nm). It was discovered that if the Al layer had a grating structure, grating-coupled SPPs and scattered light could affect the absorption spectra of the NWs. The back reflector can influence the optical characteristics of the NW devices, via reflection of both light in NWs and light between NWs as well as plasmonic field confinement.

**Figure 8:**
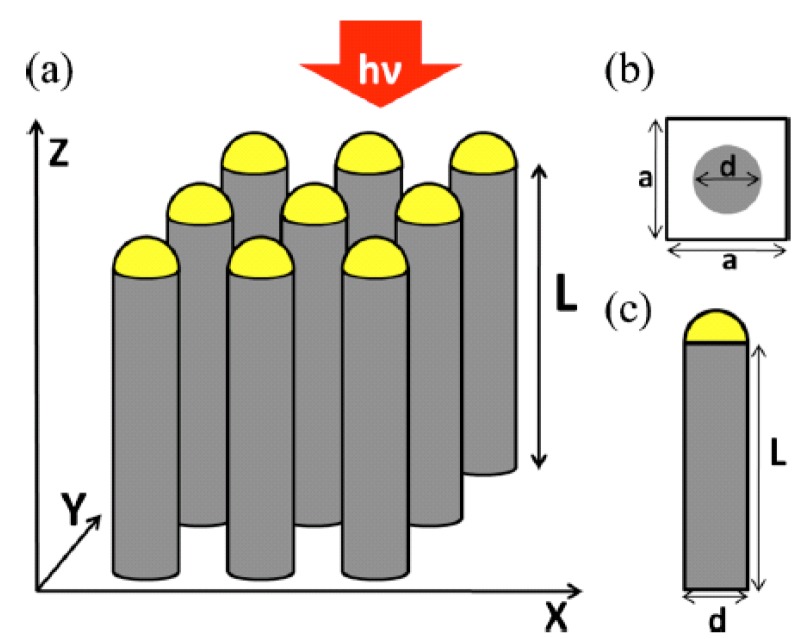
(**a**) Schematic of the Si NW array with hemispherical metal caps. (**b**) Top view of a single nanowire. (**c**) Cross-sectional view of a single nanowire. Reprinted with permission from [57]. Copyright © 2010 AIP Publishing LLC.

Aside from the plasmonic layer located on the backside of the semiconductor wire arrays, the function of plasmonic NPs in wire array-based photovoltaic devices is also discussed. In 2010, Lin and Povinelli [57] studied the effects of metallic caps on top of vertically aligned silicon nanowire arrays (Figure 8). They simulated how the diameter and spacing of the nanowires and the composition of the metal caps affect integrated absorption across the solar spectrum, and drew the conclusion that, for a wide range of nanowire diameters and spacings, metallic caps degrade the integrated absorption in the photoactive region. Although plasmonic enhancement occurs in thin-film photovoltaic systems, metallic caps on top of the Si nanowire array do not increase the efficiency in the case of nanowire arrays. In 2011, we discussed the plasmonic effect of metallic NPs coated onto the sidewalls of Si NWs [58]. Au NP-coated Si NW arrays predicted high absorption enhancement in theory. However, no such result is observed experimentally. We believe that may be because of the high intrinsic loss in metallic NPs. According to the efforts of Kelzenberg [59], dielectric NPs such as Al_2_O_3_ could efficiently scatter light that might otherwise pass between the wires other than metallic NPs.

## 4. Surface Plasmonics in Single Nanowire Solar Cells

Radial p–n junction [60] single nanowire solar cells (SNSCs) are a promising candidate for photovoltaic devices due to the occurrence of leaky mode resonance (LMR) [61]. To date, SNSCs have been realized by using a variety of active semiconductors. Several types of theoretical and experimental work have been done [62,63,64,65,66,67]. By tuning the size of single nanowires, the LMRs in the nanowires could be optimized for photovoltaic cells. In addition, to further enhance the absorption of sunlight, metallic nanostructures are conceived to be combined into solar cells. Regarding the implementation of metallic SPs, there are two main geometries. The first is to use plasmonic NPs to couple the incident light or generate near-field SPs [63,64,65]; the second is to use a metallic core embedded into a single nanowire [66,67].

**Figure 9:**
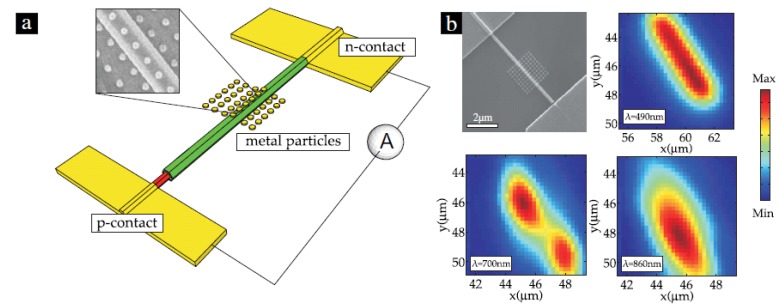
(**a**) Illustration of a contacted radial p–i–n junction GaAs NW. (**b**) A SEM image of a typical device is shown in the top left corner of the picture. The other images show examples of photocurrent maps. Reprinted with permission from [64]. Copyright © IOP Publishing Ltd and Deutsche Physikalische Gesellschaft.

In 2010, Brittman *et al.*, studied the effect of octahedral silver nanocrystals on the absorption of a silicon nanowire solar cell [63]. Increases in the nanowire’s absorption and photocurrent arose from the coupling of the nanocrystal’s dipolar and quadrupolar resonances to the wire. Decreases occurred at wavelengths for which the particle perturbs the resonances of the nanowire itself. For isolated nanocrystals, simulations and scanning photocurrent mapping both indicated that the observed increases in photocurrent arose from the nanocrystal and resulted from both near-field interactions and far-field scattering. In 2011, Colombo *et al.*, reported their findings on the optical properties of a contacted radial p–i–n junction GaAs NW-decorated with metal NPs [64]. Light absorption in NWs through LMRs can be further engineered by choosing the appropriate geometry of the substrate and interaction with SP modes on the metallic NPs. As an example, they showed that by placing NPs on different facets and by controlling the NP-to-NW distance with a spacer oxide, the NW absorption near the band gap energy can be increased by a factor of 5. Figure 9 illustrates a contacted radial p–i–n junction GaAs NW, and the experimental scanning electron microscope (SEM) image with simulation photocurrent maps at different wavelengths. Their experimental data well matches the simulation results, giving new degrees of freedom for engineering light absorption in NWs for the applications in solar cells as well as in photo-detectors. In 2014, Robak *et al.*, reported the absorption enhancement in a single Si NW caused by a metallic bowtie nanoantenna as a function of geometric parameters of the system [65]. The large local near-field around the bowtie’s vertexes caused by localized surface plasmon resonance of the metallic structure was proven to be the source of the enhancement.

**Figure 10:**
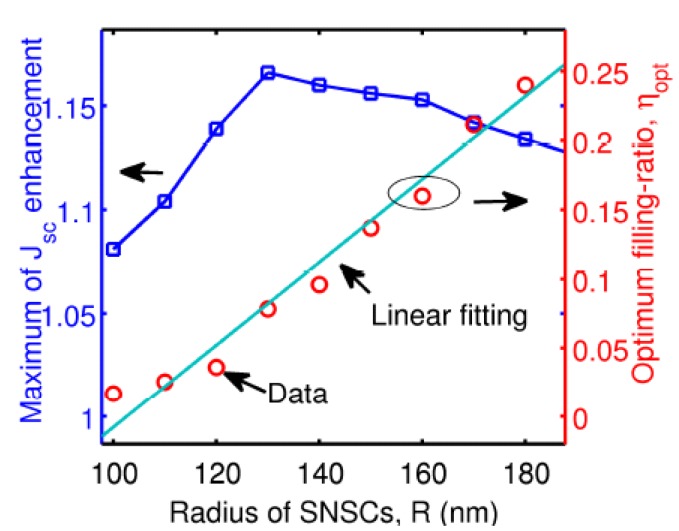
Maximum *J*_sc_ enhancement (blue squares) and optimum filling ratio (red circles) as a function of the radius of single nanowire solar cells (SNSCs). The light green line is the linear fitting for the relationship between η_opt_ and *R*. Reprinted with permission from [66]. Copyright © 2012 Optical Society of America.

In the research field of single semiconductor NWs with a metallic core, Zhan *et al.*, demonstrated numerically the function of metallic-cores in single a-Si nanowire solar cells in 2010 [66]. Both p-polarized and s-polarization incident light were considered. Their simulation results suggested that most LMRs couple into localized surface plasmons or disappear under p-polarized illumination, yet are not affected much by Mie scattering under s-polarization. The contributors to light trapping were demonstrated to be mainly LSPs, followed by Mie scattering and LMRs. A detailed geometric parameter sweep was performed. Figure 10 shows the maximum short circuit current density enhancement (square points in blue) and its optimum filling-ratio (circle points in red) as a function of the radius of SNSCs. The optimized filling ratio increases almost linearly with the radius of SNSCs, which is due to a combination effect of shifts in LMR and LSP resonances. An enhancement of 16.6% in photocurrent was produced by SNSCs with a nanowire radius of 130 nm and a core radius of 36 nm. The influence of Ag core position on enhanced photon absorption of single a-Si NW solar cells was reported by Shi *et al.*, in 2013 [67]. The optimized location of Ag core could be theoretically predicted at a certain diameter of a-Si NW. The study suggests that an off-center core could be better a co-axial core-shell nanostructure.

## 5. Surface Plasmonics-Induced Hot-Electron Generation

Recently, there has been increasing interest in utilizing plasmonic nanostructures to directly convert collected light into electrical energy by generating hot electrons [68,69,70]. Once photons are absorbed in the nanostructures, LSPR can occur, and confined free electrons oscillate with the same frequency as the incident radiation. Meanwhile, plasmons can decay, transferring the accumulated energy to electrons in the conduction band of the material. This process produces highly energetic electrons, also known as hot electrons. Those hot electrons can escape from plasmonic nanostructures and be collected by a contacted semiconductor, thereby forming a metal–semiconductor Schottky junction. For example, surface plasmons in Au and Ag nanostructures can transfer energies between approximately 1 eV and 4 eV to hot electrons. If those hot electrons could be efficiently extracted from the metal via internal photoemission (IPE) across a metal-semiconductor Schottky junction, this could open up an alternative photocurrent mechanism for solar cells.

**Figure 11:**
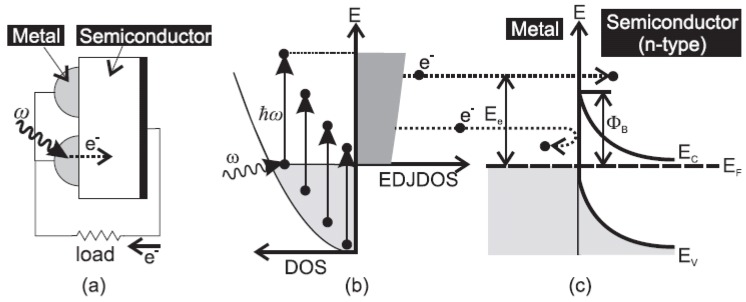
(**a**) Geometry of a plasmon-enhanced internal photoemission solar cell. (**b**) Excitation of electrons in the metal from occupied energy levels in the conduction band (shaded gray) to unoccupied levels above the Fermi energy *E*_F_. (**c**) Energy diagram of the Schottky junction at the metal-semiconductor interface (shown for an n-type semiconductor). Hot electrons with energy > Φ_B_ can be emitted over the barrier into the semiconductor; those without enough energy are reflected back into the metal. Reprinted with permission from [70]. Copyright © 2012 AIP Publishing LLC.

In 2012, White and Catchpole studied the theoretical efficiency limits when considering the plasmon-enhanced IPE in metal-semiconductor Schottky junction solar cells [70]. Figure 11a shows the basic cell geometry, consisting of a metal nanoparticle absorber on a semiconductor surface. Figure 11b demonstrates schematic excitation of electrons in the metal from occupied energy levels in the conduction band (shaded gray) to unoccupied levels above the Fermi level. Figure 11c shows the energy diagram of the Schottky junction at the metal-semiconductor interface. The authors considered the four steps of photocurrent generation to be (i) absorption of photons into the metal and generation of hot electrons; (ii) ballistic transport of hot electrons through the metal to the interface; (iii) emission of electrons across the junction; and (iv) collection of electrons at a contact. Although only ~7% maximum of a theoretical efficiency is predicted by the use of realistic materials, the conversion efficiency might be possible to reach as high as 22.6% if the density of the sates (DOS) of the light absorbers is properly modified.

## 6. Conclusions

In this review, we summarized recent developments on surface plasmons in photovoltaic applications, including both inorganic and organic thin film solar cells, semiconductor nanowire/microwire array solar cells, single nanowire solar cells, as well as solar cells that utilize a SP-induced hot electron generation effect. SPs have been implemented in solar cells in the form of NPs with a variety of shapes, materials and sizes. The position and density of NPs in the solar cell could significantly influence photovoltaic performance. Metallic nanogratings, plasmonic nanosurfaces, nanohole arrays, and metamaterial plasmonic absorber structures can also be designed and optimized for higher efficiency solar cells. Metallic-core semiconductor-shell single wire solar cells played an important role in the development of plasmon solar cells. In addition, a new solar energy conversion scheme related to the SP-induced hot electron generation effect opens up a way to create photovoltaic devices.
